# Machine Learning–Based Survival Prediction Tool for Adrenocortical Carcinoma

**DOI:** 10.1210/clinem/dgaf096

**Published:** 2025-02-14

**Authors:** Emre Sedar Saygili, Yasir S Elhassan, Alessandro Prete, Juliane Lippert, Barbara Altieri, Cristina L Ronchi

**Affiliations:** Division of Endocrinology and Metabolism, Department of Internal Medicine, Faculty of Medicine, Canakkale Onsekiz Mart University, Canakkale 17020, Turkey; Department of Metabolism and Systems Science, College of Medicine and Health, University of Birmingham, Birmingham B15 2TT, UK; Department of Metabolism and Systems Science, College of Medicine and Health, University of Birmingham, Birmingham B15 2TT, UK; Department of Endocrinology, Queen Elizabeth Hospital Birmingham, University Hospitals Birmingham NHS Foundation Trust, Birmingham B15 2GW, UK; Department of Metabolism and Systems Science, College of Medicine and Health, University of Birmingham, Birmingham B15 2TT, UK; Centre for Endocrinology, Diabetes and Metabolism, Birmingham Health Partners, Birmingham B15 2TT, UK; Department of Endocrinology, Queen Elizabeth Hospital Birmingham, University Hospitals Birmingham NHS Foundation Trust, Birmingham B15 2GW, UK; NIHR Birmingham Biomedical Research Centre, University of Birmingham and University Hospitals Birmingham NHS Foundation Trust, Birmingham B15 2TH, UK; Institute of Human Genetics, University of Wuerzburg, 97070 Wuerzburg, Germany; Division of Endocrinology and Diabetes, Department of Internal Medicine I, University Hospital, University of Wuerzburg, 97080 Wuerzburg, Germany; Department of Metabolism and Systems Science, College of Medicine and Health, University of Birmingham, Birmingham B15 2TT, UK; Centre for Endocrinology, Diabetes and Metabolism, Birmingham Health Partners, Birmingham B15 2TT, UK; Department of Endocrinology, Queen Elizabeth Hospital Birmingham, University Hospitals Birmingham NHS Foundation Trust, Birmingham B15 2GW, UK

**Keywords:** model, adrenal cancer, mortality, prognosis, precision medicine

## Abstract

**Context:**

Adrenocortical carcinoma (ACC) is a rare, aggressive malignancy with difficult to predict clinical outcomes. The S-GRAS score combines clinical and histopathological variables (tumor stage, grade, resection status, age, and symptoms) and showed good prognostic performance for patients with ACC.

**Objective:**

To improve ACC prognostic classification by applying robust machine learning (ML) models.

**Method:**

We developed ML models to enhance outcome prediction using the published S-GRAS dataset (n = 942) as the training cohort and an independent dataset (n = 152) for validation. Sixteen ML models were constructed based on individual clinical variables. The best-performing models were used to develop a web-based tool for individualized risk prediction.

**Results:**

Quadratic Discriminant Analysis, Light Gradient Boosting Machine, and AdaBoost Classifier models exhibited the highest performance, predicting 5-year overall mortality (OM), and 1-year and 3-year disease progression (DP) with F1 scores of 0.79, 0.63, and 0.83 in the training cohort, and 0.72, 0.60, and 0.83 in the validation cohort. Sensitivity and specificity for 5-year OM were at 77% and 77% in the training cohort, and 65% and 81% in the validation cohort, respectively. A web-based tool (https://acc-survival.streamlit.app) was developed for easily applicable and individualized risk prediction of mortality and disease progression.

**Conclusion:**

S-GRAS parameters can efficiently predict outcome in patients with ACC, even using a robust ML model approach. Our web app instantly estimates the mortality and disease progression for patients with ACC, representing an accessible tool to drive personalized management decisions in clinical practice.

Adrenocortical carcinoma (ACC) is a rare endocrine malignancy with a yearly incidence of only 0.7 to 2 cases per million people and a notoriously unfavorable prognosis (1-3). The median overall survival (OS) in patients with ACC is 3 to 4 years, with wide variability reflecting the disease stage at diagnosis. For instance, the 5-year survival rates were reported as 60% to 80% for tumors confined to the adrenal bed, 35% to 50% for locally advanced disease, and 0% to 28% for metastatic disease (4). The tumor stage, according to the European Network for the Study of Adrenal Tumors (ENSAT) classification (4-6), represents the most accepted clinical prognostic factor. However, other well-recognized parameters associated with clinical outcomes are the resection (R) status of the primary tumor and the Ki67 proliferation index (4-8) both with limitations (4, 9). Our recent large international multicenter study showed that the S-GRAS score is the most powerful prognostic factor for predicting survival in patients with ACC (9). The S-GRAS score combines readily available clinical factors, age, and symptoms at presentation, with ENSAT tumor stage and the histopathological parameters R status and Ki67 to generate a more robust prediction than the individually considered parameters. By definition, the S-GRAS score is only available for adult patients who underwent surgical resection of the primary tumor.

Molecular markers derived from previous pangenomic studies (10, 11) have also been proposed to play a role as prognostic factors in ACC (12, 13). Our recent findings also indicate that the incorporation of selected targeted DNA-based biomarker assessment on routinely obtained formalin-fixed paraffin-embedded tissue samples with the S-GRAS parameters (COMBI score) significantly enhances the accuracy of prognostic evaluation for ACC, surpassing the limitations of relying solely on the S-GRAS score (14). However, these molecular markers are not widely available and have not yet been introduced in clinical practice.

Supervised machine learning (ML) is a subset of artificial intelligence that uses algorithms to automatically gain insight and recognize patterns from data leading to the generation of decision models. These algorithms have numerous promising applications in various medical fields (15). The use of ML-based tools has grown exponentially, particularly in oncology, where multiple ML algorithms have been proposed for prognostic prediction. ML is en route to be vital in every step of oncological strategies and patient management in the foreseeable future, ushering in the era of precision medicine (16, 17).

In this study, we aimed to develop accurate ML models for predicting clinical outcomes in patients with ACC after tumor resection and deploy them as a web-based decision support tool.

## Material and Methods

### Patient Cohorts

The present study is based on datasets from 2 previously published studies that investigated the role of clinical and histopathological parameters for the prognostic classification of adult patients with ACC (9, 14). The first by Elhassan et al, a multicenter project coordinated by our group on behalf of the ENSAT, demonstrated superior prognostic performance of the S-GRAS score over the currently used ENSAT tumor stage and Ki67 index (9). This study included baseline and follow-up data from 942 patients with ACC (583 F/359 M) and is defined here as the S-GRAS dataset. Each participating center provided permission to reuse their anonymized data for the present study.

**Figure 1.**
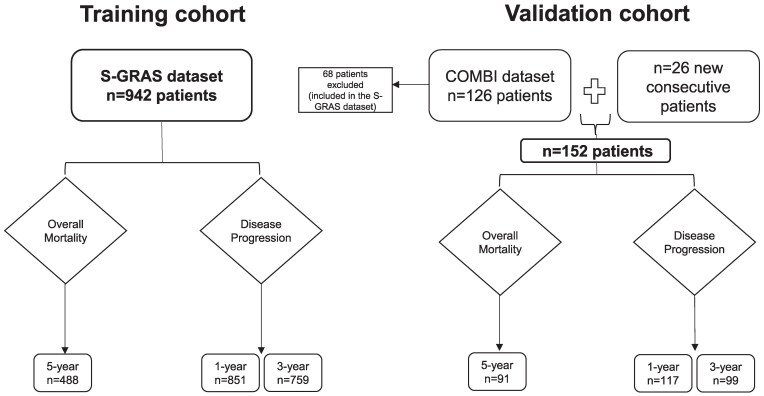
Description of number of patients with adrenocortical carcinoma (ACC) included in the training (n = 942) and validation cohorts (n = 152) and availability of outcomes (5-year overall mortality and 1-year or 3-year disease progression). New independent and consecutive patients with ACC from Queen Elizabeth Hospital Birmingham (UK).

In the second study by Lippert et al, we found that incorporating selected DNA-based biomarkers with the S-GRAS score further improved prognostication in ACC (COMBI score) (14). This study included baseline and follow-up data for 194 patients; 68 of them being excluded from this study as they were also part of the S-GRAS dataset. The remaining 126 patients are here defined as the COMBI dataset. We utilized a training and validation cohort for each outcome while creating and validating our ML models. The S-GRAS dataset was used as training cohort, while the COMBI dataset was combined with 26 newly recruited consecutive patients with ACC (18 F/8 M) from the Queen Elizabeth Hospital Birmingham (who underwent adrenalectomy 2019-2023) and used as validation cohort (n = 126 + 26 = 152). A schematic representation of the included cohorts and study protocol is provided in Fig. 1. Only adult patients aged 18 years or older have been included in the study.

The local ethics committee approved the study protocol (PrimeAct study REC 20/NW/0207, University of Birmingham). Written informed consent was obtained from all newly recruited subjects.

### Machine Learning Model Components

To build our model, we used the individual S-GRAS parameters as previously described (9), namely, ENSAT stage (1 or 2 = 0 points, 3 = 1 point, 4 = 2 points), grade (Ki67 0-9% = 0; 10-19% = 1; ≥ 20% = 2 points), R of the primary tumor (R0 = 0 points, RX = 1 point, R1 = 2 points, R2 = 3 points), age at diagnosis (less than 50 years = 0 points, 50 years or older = 1 point), and the presence of hormone, tumor, or systemic cancer-related symptoms at presentation (no = 0 points, yes = 1 point). Characteristics required to calculate the S-GRAS score were available from previous datasets and new patients with ACC. By definition, each patient was included in only 1 cohort (Fig. 1).

### Outcome Measures

The primary outcome was 5-year overall mortality (OM) defined from primary tumor resection to death or last available follow-up (death within 5 years = 1, no death within 5 years = 0). Secondary outcomes included 1-year disease progression (DP) and 3-year DP defined from primary tumor resection to the first radiological evidence of progression or last available follow-up (eg, disease relapse in patients after radical resection or progressive and/or new lesions in patients with advanced disease, as defined by local radiologists according to the RECIST 1.1 criteria). A schematic representation of the included cohorts and study outcomes is provided in Fig. 1.

### Model Performance

The performance of the models was evaluated using multiple metrics, including the area under the receiver operating characteristic curve (AUC), accuracy, precision, sensitivity (recall), specificity, and F1 score. The model with the highest F1 score was considered the top-performing model. We determined the contribution of each feature to the top model using SHapley Additive exPlanations (SHAP) values (18).

### Computational Methods and Statistical Analysis

We utilized the PyCaret 3.1 library, a user-friendly ML framework in Python, accessible at https://pycaret.gitbook.io (accessed on February 4, 2024). This high-level library, which incorporates Numpy, Pandas, and Scikit-learn, simplifies outcomes classification by automating various tasks such as data preprocessing, feature engineering, and model selection during the model development and evaluation process (19). Sixteen supervised ML models were created using 10-fold cross-validation. These ML models were Decision Tree, Extra Trees, Random Forest, K Neighbors, Quadratic Discriminant Analysis, Logistic Regression, Ada Boost Classifier, Linear Discriminant Analysis, Ridge Classifier, Dummy Classifier, SVM-Linear Kernel, Extreme Gradient Boosting, Light Gradient Boosting, Gradient Boosting, CatBoost Classifier, and Naive Bayes. The F1 score is calculated as the harmonic mean of sensitivity and precision. The sixteen models were rigorously evaluated in the training cohort using 10-fold cross-validation (Fig. S1 (20)), and the top-performing model for each outcome was chosen by F1 score. The data for the training cohort were provided as the average of 10 different results. This approach aimed to prevent the models from overfitting.

Normally distributed continuous variables are represented by mean ± standard deviation, while non-normally distributed variables are expressed using the median and interquartile range (IQR). Categorical variables are presented as numbers and percentages. Normally and non-normally distributed continuous variables were compared using the Student t-test and Mann–Whitney U test, respectively. Categorical variables were compared using Pearson's chi-square test. Classical statistical analysis was performed with Jamovi (Version 2.3) (21). *P* < .05 was considered statistically significant.

Streamlit, a Python library, serves as a powerful tool for deploying ML models as interactive web applications, accessible at https://github.com/streamlit/streamlit (accessed on February 4, 2024). Streamlit was used for deploying the models as an online survival tool website (https://acc-survival.streamlit.app).

## Results

### Baseline Characteristics and Outcomes

The S-GRAS dataset was used as training cohort (n = 942), while the COMBI dataset combined with newly-recruited consecutive patients with ACC was used as validation cohort (n = 152). The study assessed a total of 579, 968, and 858 patients’ data for 5-year OM, 1-year DP, and 3-year DP, respectively (Fig. 1). Considering the training and validation cohorts together, the 5-year OM, 1-year DP, and 3-year DP rates were 55.1%, 39.7%, and 67.6%, respectively. There were no significant differences in the clinical and histopathological characteristics between the training and the validation cohorts for the evaluated outcomes except for the ENSAT stage at 1-year DP. Details about the patients’ characteristics within the different outcome groups are shown in Table 1.

**Table 1.** Clinical and histopathological characteristics of patients with adrenocortical carcinoma (ACC) within outcome groups

	5-year overall mortality				1-year disease progression				3-year disease progression			
	Training (n = 488)	Validation (n = 91)	Total (n = 579)	*P* value	Training (n = 851)	Validation (n = 117)	Total (n = 968)	*P* value	Training (n = 759)	Validation (n = 99)	Total (n = 858)	*P* value
Age				.580				.825				.548
<50-year-old	242 (49.6%)	48 (52.7%)	290 (50.1%)		423 (49.7%)	60 (51.3%)	483 (49.9%)		382 (50.3%)	53 (53.5%)	435 (50.7%)	
≥50-year-old	246 (50.4%)	43 (47.3%)	289 (49.9%)		428 (50.3%)	57 (48.7%)	485 (50.1%)		377 (49.7%)	46 (46.5%)	423 (49.3%)	
Symptoms at presentation				.080				.192				.072
No	128 (26.2%)	32 (35.2%)	160 (27.6%)		251 (29.5%)	42 (35.9%)	293 (30.3%)		206 (27.1%)	36 (36.4%)	242 (28.2%)	
Yes	360 (73.8%)	59 (64.8%)	419 (72.4%)		600 (70.5%)	75 (64.1%)	675 (69.7%)		553 (72.9%)	63 (63.6%)	616 (71.8%)	
ENSAT stage				.417				.**016**				.389
1-2	262 (53.7%)	43 (47.3%)	305 (52.7%)		504 (59.2%)	54 (46.2%)	558 (57.6%)		427 (56.3%)	49 (49.5%)	476 (55.5%)	
3	120 (24.6%)	28 (30.8%)	148 (25.6%)		198 (23.3%)	40 (34.2%)	238 (24.6%)		187 (24.6%)	30 (30.3%)	217 (25.3%)	
4	106 (21.7%)	20 (22.0%)	126.0 (21.8%)		149 (17.5%)	23 (19.7%)	172 (17.8%)		145 (19.1%)	20 (20.2%)	165 (19.2%)	
Resection status				.104				.073				.349
R0	318 (65.2%)	66 (72.5%)	384 (66.3%)		588 (69.1%)	80 (68.4%)	668 (69%)		505 (66.5%)	72 (72.7%)	577 (67.2%)	
RX	47 (9.6%)	12 (13.2%)	59 (10.2%)		93 (10.9%)	20 (17.1%)	113 (11.7%)		88 (11.6%)	11 (11.1%)	99 (11.5%)	
R1	31 (6.4%)	5 (5.5%)	36 (6.2%)		48 (5.6%)	8 (6.8%)	56 (5.8%)		46 (6.1%)	7 (7.1%)	53 (6.2%)	
R2	92 (18.9%)	8 (8.8%)	100 (17.3%)		122 (14.3%)	9 (7.7%)	131 (13.5%)		120 (15.8%)	9 (9.1%)	129 (15%)	
Ki67				.333				.433				.424
0-9%	122 (25%)	23 (25.3%)	145 (25%)		234 (27.5%)	30 (25.6%)	264 (27.3%)		186 (24.5%)	25 (25.3%)	211 (24.6%)	
10-19%	97 (19.9%)	24 (26.4%)	121 (20.9%)		194 (22.8%)	33 (28.2%)	227 (23.5%)		174 (22.9%)	28 (28.3%)	202 (23.5%)	
≥20%	269 (55.1%)	44 (48.4%)	313 (54.1%)		423 (49.7%)	54 (46.2%)	477 (49.3%)		399 (52.6%)	46 (46.5%)	445 (51.9%)	
Outcome				.794				.103				.414
No	218 (44.7%)	42 (46.2%)	260 (44.9%)		522 (61.3%)	62 (53%)	584 (60.3%)		250 (32.9%)	28 (28.3%)	278 (32.4%)	
Yes	270 (55.3%)	49 (53.8%)	319 (55.1%)		329 (38.7%)	55 (47%)	384 (39.7%)		509 (67.1%)	71 (71.7%)	580 (67.6%)	

Significant *P* values are in bold.

Abbreviations: R0, No residual tumor; R1, microscopic residual tumor; R2, macroscopic residual tumor; RX, presence of residual tumor cannot be assessed.

### ML Models of 5-Year Overall Mortality (Primary Outcome)

Quadratic discriminant analysis was the top model for 5-year OM in the training cohort with an achieved accuracy of 0.77, with an AUC of 0.85. Both sensitivity and specificity were recorded at 0.77. The F1 score was calculated at 0.79. The scores for 5-year OM in the training cohort are shown for all ML models in Table 2. The model with the highest F1 score was chosen as the top-performing model, even though several models demonstrated an AUC greater than 0.81. In the validation cohort, the quadratic discriminant analysis exhibited sensitivity of 65%, specificity of 81%, AUC of 0.79, and F1 score of 0.72.

**Table 2.** Machine learning models for 5-year overall mortality in the training cohort (n = 488)

Model	Accuracy	AUC	Sensitivity	Precision	F1	Kappa	MCC	Specificity
Quadratic Discriminant Analysis	0.77	0.85	0.77	0.80	0.79	0.53	0.54	0.77
Logistic Regression	0.76	0.85	0.78	0.79	0.78	0.52	0.52	0.74
Ridge Classifier	0.76	0.00	0.79	0.78	0.78	0.51	0.51	0.72
Ada Boost Classifier	0.75	0.84	0.81	0.76	0.78	0.49	0.50	0.68
Linear Discriminant Analysis	0.76	0.85	0.79	0.78	0.78	0.51	0.51	0.72
CatBoost Classifier	0.75	0.84	0.80	0.76	0.78	0.48	0.49	0.68
Decision Tree Classifier	0.75	0.80	0.77	0.77	0.77	0.49	0.49	0.71
SVM—Linear Kernel	0.73	0.00	0.80	0.75	0.77	0.45	0.47	0.65
Random Forest Classifier	0.74	0.82	0.80	0.75	0.77	0.47	0.48	0.67
Extra Trees Classifier	0.75	0.81	0.78	0.77	0.77	0.49	0.50	0.71
Extreme Gradient Boosting	0.74	0.82	0.80	0.75	0.77	0.47	0.48	0.67
Light Gradient Boosting Machine	0.74	0.84	0.80	0.75	0.77	0.46	0.47	0.66
K Neighbors Classifier	0.73	0.81	0.80	0.74	0.76	0.44	0.45	0.64
Gradient Boosting Classifier	0.73	0.83	0.77	0.76	0.76	0.45	0.46	0.69
Naive Bayes	0.74	0.85	0.61	0.88	0.71	0.49	0.52	0.89
Dummy Classifier	0.55	0.50	1.00	0.55	0.71	0.00	0.00	0.00

Abbreviations: AUC, area under curve; F1, harmonic mean of the precision and sensitivity; MCC, Matthews correlation coefficient; SVM, support vector machine.

### ML Models of 1-Year and 3-Year DP (Secondary Outcomes)

In the training cohort, the Ada Boost Classifier (ABC) proved to be the superior model for predicting 3-year DP. ML models scores for 3-year DP training cohort are shown in Table 3. ABC exhibited an F1 score of 0.83 and an AUC of 0.79. Notably, ABC achieved a sensitivity of 88% but showed a specificity of 50%. Conversely, the Naïve Bayes model displayed a higher specificity of 71%; however, it yielded lower accuracy and F1 score than ABC (0.71, 0.76 vs 0.76, 0.83, respectively). In the validation cohort, the ABC exhibited sensitivity of 79%, specificity of 71%, AUC of 0.87, and F1 score of 0.83.

**Table 3.** Machine learning models for 3-year and 1-year disease progression in the training cohort (n = 942)

Model	Accuracy	AUC	Sensitivity	Precision	F1	Kappa	MCC	Specificity
**3-year disease progression**								
Ada Boost Classifier	0.76	0.79	0.88	0.78	0.83	0.41	0.42	0.50
Logistic Regression	0.75	0.80	0.88	0.78	0.82	0.40	0.41	0.50
SVM—Linear Kernel	0.75	0.00	0.85	0.80	0.82	0.41	0.42	0.54
Ridge Classifier	0.75	0.00	0.87	0.78	0.82	0.40	0.40	0.50
Linear Discriminant Analysis	0.74	0.79	0.86	0.78	0.82	0.39	0.39	0.50
Gradient Boosting Classifier	0.73	0.77	0.85	0.77	0.81	0.34	0.35	0.48
K Neighbors Classifier	0.73	0.76	0.80	0.80	0.80	0.40	0.40	0.59
Quadratic Discriminant Analysis	0.74	0.79	0.80	0.82	0.80	0.43	0.43	0.64
Light Gradient Boosting Machine	0.72	0.77	0.84	0.76	0.80	0.33	0.34	0.48
Dummy Classifier	0.67	0.50	1.00	0.67	0.80	0.00	0.00	0.00
Decision Tree Classifier	0.71	0.74	0.81	0.77	0.79	0.32	0.33	0.50
Random Forest Classifier	0.71	0.75	0.82	0.76	0.79	0.32	0.32	0.49
Extra Trees Classifier	0.71	0.74	0.81	0.77	0.79	0.32	0.32	0.49
Extreme Gradient Boosting	0.70	0.75	0.82	0.76	0.79	0.30	0.31	0.46
CatBoost Classifier	0.71	0.77	0.83	0.76	0.79	0.30	0.30	0.45
Naive Bayes	0.71	0.79	0.70	0.83	0.76	0.39	0.40	0.71
**1-year disease progression**								
Light Gradient Boosting Machine	0.74	0.78	0.57	0.70	0.63	0.43	0.43	0.84
Quadratic Discriminant Analysis	0.74	0.79	0.54	0.72	0.62	0.43	0.44	0.86
Gradient Boosting Classifier	0.73	0.77	0.57	0.69	0.62	0.42	0.42	0.84
Extra Trees Classifier	0.74	0.76	0.53	0.74	0.62	0.43	0.45	0.88
K Neighbors Classifier	0.72	0.77	0.56	0.67	0.61	0.39	0.40	0.82
Decision Tree Classifier	0.74	0.76	0.52	0.74	0.61	0.42	0.44	0.88
Random Forest Classifier	0.73	0.77	0.54	0.71	0.61	0.41	0.42	0.85
Ada Boost Classifier	0.73	0.78	0.55	0.70	0.61	0.41	0.42	0.85
Extreme Gradient Boosting	0.73	0.77	0.55	0.71	0.61	0.42	0.43	0.85
Logistic Regression	0.74	0.78	0.52	0.73	0.60	0.42	0.43	0.88
Naive Bayes	0.73	0.79	0.52	0.72	0.60	0.41	0.42	0.87
Ridge Classifier	0.74	0.00	0.52	0.73	0.60	0.41	0.43	0.88
Linear Discriminant Analysis	0.74	0.78	0.52	0.72	0.60	0.41	0.43	0.87
Light Gradient Boosting Machine	0.74	0.78	0.57	0.70	0.63	0.43	0.43	0.84
Quadratic Discriminant Analysis	0.74	0.79	0.54	0.72	0.62	0.43	0.44	0.86
Gradient Boosting Classifier	0.73	0.77	0.57	0.69	0.62	0.42	0.42	0.84
CatBoost Classifier	0.73	0.77	0.54	0.69	0.60	0.40	0.41	0.84
SVM—Linear Kernel	0.66	0.00	0.36	0.52	0.40	0.22	0.24	0.85
Dummy Classifier	0.61	0.50	0.00	0.00	0.00	0.00	0.00	1.00

Abbreviations: AUC, area under curve; F1, harmonic mean of the precision and sensitivity; MCC, Matthews correlation coefficient; SVM, support vector machine.

The Light Gradient Boosting Machine was the top model for 1-year DP training cohort. The Light Gradient Boosting Machine exhibited sensitivity of 57%, specificity of 84%, AUC of 0.78, and F1 score of 0.63 in the training cohort. ML models scores for 1-year DP training cohort are presented in Table 3.

### Performance of ML Models in the Training and Validation Cohorts

A summary of the top-performing models for outcomes and their performance scores in the validation cohort is shown in Table 4. In particular, sensitivity and specificity for 5-year OM were 77% and 77% in the training cohort, and 65% and 81% in the validation cohort, respectively. The sensitivity and specificity for 3-year DP were 88% and 50% in the training cohort, and 79% and 71% in the validation cohort, respectively. The sensitivity and specificity for 1-year DP were 57% and 84% in the training cohort, and 53% and 81% in the validation cohort, respectively. The SHAP plots for the ML models are presented in Figs. S2-4 (20).

**Table 4.** Performance of the best machine learning models for each outcome in the training (n = 942) and validation cohorts (n = 152)

Outcomes	Best model	Cohort	Accuracy	AUC	Sensitivity	Precision	F1	Specificity
5-year overall mortality	Quadratic Discriminant Analysis	Training	0.77	0.85	0.77	0.80	0.79	0.77
		Validation	0.73	0.79	0.65	0.80	0.72	0.81
3-year disease progression	Ada Boost Classifier	Training	0.76	0.79	0.88	0.83	0.83	0.50
		Validation	0.77	0.87	0.79	0.83	0.83	0.71
1-year disease progression	Light Gradient Boosting Machine	Training	0.74	0.78	0.57	0.70	0.63	0.84
		Validation	0.68	0.74	0.53	0.71	0.60	0.81

Abbreviations: AUC, area under curve; F1, harmonic mean of the precision and sensitivity.

### Interactive Web Application

We also aimed to deploy the 3 top-performing ML prediction models (Quadratic Discriminant Analysis, Light Gradient Boosting Machine, and Ada Boost Classifier) as a web-based decision support tool for clinicians (https://acc-survival.streamlit.app). Specifically, Streamlit in Python was used for deploying the models as an interactive website. Our web application provides a numerical probability for clinical outcomes for adult patients with ACC (ie, risk of mortality and DP) ranging from 0% to 100%. The web app is free to use and user-friendly, therefore easy to implement in clinical practice (Fig. S5 (20)). It is aimed to allow clinicians and qualified healthcare staff to quickly calculate probabilities of death and DP by inputting readily available S-GRAS parameters (ie, age, symptoms at diagnosis, ENSAT stage, resection status, and Ki67 values).

## Discussion

ACC is a rare aggressive disease with a generally poor—but difficult to predict—prognosis and limited therapeutic options (22). Therefore, optimizing the management strategies for patients with ACC to prevent or slow down disease recurrence or progression for as long as possible is crucial. To this end, it is important to accurately identify patients at high-risk disease recurrence after the surgical resection of the primary tumor. Current tools used for risk stratification of patients with ACC are however inadequate. Predictive tools, such as those proposed in this study, can be used to guide clinicians customize strategies for monitoring cancer recurrence (eg, frequency of radiological surveillance) and make individualized treatment decisions (eg, adjuvant and local therapies).

Hereby, using multiple ML models, we clearly validated that S-GRAS parameters—previously established within a European multicenter study (9)—can effectively predict OM and DP in ACC. Moreover, we deployed our top models to create a web-based application that instantly calculates the “probability of outcomes” (ie, the risk of death and DP) for patients with ACC after tumor resection based on the readily available S-GRAS parameters. To our knowledge, this is the first ML-based online survival prediction tool for ACC.

By comparing sixteen ML methods, the top-performing models to predict 5-year OM and DP for ACC were selected. To test the prognostic performance of S-GRAS parameters, we used 2 large cohorts of patients with ACC, namely, a training cohort (n = 942) (9) and a validation cohort (n = 152) (14). Of note, we could demonstrate that sensitivity and specificity for 5-year OM were good in both cohorts (ie, 77% and 77% in the training cohort and 65% and 81% in the validation cohort, respectively).

In a previous study, we tested the prognostic role of the COMBI score, obtained by merging S-GRAS parameters with 2 DNA-based biomarkers (ie, alterations in Wnt/β-catenin and Rb/p53 pathways and hypermethylated PAX5) (14). The COMBI score showed a higher discriminative prognostic model than the S-GRAS score, with a Harrell's C index of 0.724 and 0.765 for OS, and 0.717 and 0.670 for progression-free survival, respectively (14). In both these studies, we used Cox regression models for survival analysis by including censored data in survival models. However, in our present study, we did not include censored data, and instead evaluated the results as binary (present/absent) and analyzed the 5-year OM and DP status in years 1 and 3. Therefore, it is not be possible to make a direct, comprehensive comparison with previous studies. While the AUC is more commonly used in binary outcome scenarios, Harrell's C-index is specifically designed for right-censored survival outcomes. Both metrics serve as valuable tools for comparing the predictive power of different models, with Harrell's C-index being particularly useful in scenarios involving censored data (23). As we defined patient outcomes using a binary value in our studies, Harrell's C-index is equivalent to the AUC (24-26). COMBI and S-GRAS Cox regression–based models have higher Harrell's C-index of 0.765, but for ML-based S-GRAS the 5-year OM AUC ranges from 0.85 to 0.79. Herein, the ML-based model has higher predictive scores than our previous models.

Only a few studies have previously investigated the prognostic role of clinical parameters in ACC using ML. Kim et al (27) constructed a nomogram for predicting recurrence-free survival using data from 148 patients with ACC. The nomogram was derived by selecting 5 clinical parameters: tumor size, lymph node involvement, tumor stage, capsular infiltration, and adrenocortical hormone excess. Their model's discriminative ability for recurrence-free survival and OS showed a Harrell's C-index of 0.74 for recurrence-free survival and 0.70 for OS. We demonstrate that our models have higher predictive scores. Tang et al (28) used 4 ML models to predict survival in 825 patients with ACC diagnosed between 1975 and 2018, based on the Surveillance, Epidemiology, and End Results (SEER) dataset. The highest AUCs for predicting 5-year survival status were 0.89 and 0.87 in the training and test sets, respectively. The authors did not provide other ML model metrics, which are necessary for accurate comparison of the ML models. To date, most studies on ML models in ACC attempted to build models using a small number of patients, precluding impactful conclusions. Our entire cohort includes an exceptionally high number of patients with ACC considering the rarity of the disease.

The application of ML models in the context of S-GRAS parameters is a novel and robust approach. These models, by leveraging metrics such as accuracy, precision, sensitivity, or AUC-ROC, can effectively gauge the forecasting potential of these parameters. In comparison to traditional statistical techniques, ML models demonstrate a superior ability to identify complex, nonlinear relationships between S-GRAS parameters and clinical outcomes. This unique capability, which simpler models may overlook, enhances their predictive power. While a direct comparison may not be feasible, the use of performance metrics can provide valuable insights into the improvement of ML models. For instance, if these models demonstrate higher accuracy or superior performance on validation datasets compared to previous models, it indicates a significant enhancement in their predictive capabilities. Moreover, the use of OM, 1-year DP, and 3-year DP as metrics for patient follow-up is beneficial, as they have distinct clinical interpretations.

Medical professionals often find it difficult to perform statistical calculations, especially during clinical consultations. To enhance accessibility, we created a risk prediction system that is freely available online. Our approach is based on the top-performing ML models that can rapidly analyze minimal patient data and are easily accessed on computers and smartphones. We included a limited number of readily available variables in our model—based on the S-GRAS parameters—to reduce the effort of measuring and inputting patient data. Clinicians treating patients with ACC could use our web-based application in clinical practice to support and drive personalized management decisions.

### Study Limitations

The present study aimed to create a model based on minimal clinical parameters, useful in daily clinical practice. The total number of patients included in the training and validating cohort might be limited in general terms, but is exceptionally high considering the rarity of the disease. This includes a slight preponderance of females in agreement with the epidemiology of ACC.

The addition of DNA-based molecular alterations, including somatic variants in specific pathways and hypermethylation in PAX5 to the model was not possible due to the limited number of patients in the COMBI dataset. However, the most powerful prognostic histopathological variable, Ki67, was included. In future studies, the addition of DNA-based biomarkers or images from histopathological preparations or radiological scans could be proposed. This requires complex ML models that are less applicable in routine healthcare settings (29). The inclusion of genetic alterations, such as germline or somatic variants, including those related to genetic syndromes like Li–Fraumeni or Lynch, was not feasible. While molecular prognostic data were available for the COMBI validation cohort, they were absent from the training cohort, limiting their integration into the model. Future studies could focus on incorporating molecular features to enhance the tool's predictive capabilities.

The model does not incorporate the mENSAT classification criteria (30) (ie, additional prognostic factors for stage III-IV ACCs). These criteria are not included in the original S-GRAS score and were not widely available across the multicenter datasets used during model development. Retraining the model with mENSAT features is a valuable avenue for future refinement.

Although the current model was validated on an independent cohort unseen during training, external validation using datasets from independent clinical centers is essential to ensure its generalizability. Plans are underway to conduct such validation in subsequent studies.

ML models are capable of making predictions solely based on the datasets on which they are trained. Centers that provided data for included datasets are experienced in the management of ACC. Regular patient follow-up, effective management of medical therapy, and implementation of surgical interventions when necessary, are critically important to optimize survival in ACC. The outputs of the model are intended to provide clinicians with a guiding tool and to assist in determining follow-up frequencies. However, it is essential to consider these limits when interpreting the model's results.

Despite these limitations, the study provides an important step in developing a ML-based survival prediction tool for ACC, which may aid in clinical decision-making and personalized patient care.

### Conclusions

The S-GRAS parameters can efficiently predict clinical outcomes in patients with ACC using a robust ML model approach. To our knowledge, we provide the first ML-based survival tool for ACC. Our web app instantly estimates the mortality and DP for patients with ACC (https://acc-survival.streamlit.app). This is an accessible and readily utilizable approach in clinical practice to drive personalized management decisions (eg, to support decisions regarding adjuvant treatments and frequency of surveillance).

## Data Availability

Some or all datasets generated during and/or analyzed during the current study are not publicly available but are available from the corresponding author on reasonable request.

## Abbreviations

ABC: AdaBoost Classifier
ACC: adrenocortical carcinoma
AUC: area under the receiver operating characteristic curve
DP: disease progression
ENSAT: European Network for the Study of Adrenal Tumors
ML: machine learning
OM: overall mortality
OS: overall survival
R: resection
S-GRAS: stage, grade, resection status, age, and symptoms
